# New Inventions

**Published:** 1896-06

**Authors:** 


### NEW INVENTIONS.

An animal-poke, consisting of a wooden yoke adjusted to the neck, and to which is jointed a bar with projections; the bar extending to the knee and fastened in a loop attached to a strap secured to the forearm just above the knee.

An arrangement worked by a handle adjacent to the seat of the driver by which in the event of a runaway or accident the horse and shafts may be freed from the vehicle, and by the same lever the latter guided to a standstill.

A wedge-shaped snap-hook for fastening rein, containing a bolt which is held in place by a spring.

A hitching device, consisting of a pedal pendant from the body of the wagon, and so arranged as to grasp the ground or earth, and a hitching-rein carried from this device to the ring of the bridle.

A removable horseshoe-calk, consisting of a threaded spur fastened in threaded openings of the shoes, and secured by a washer.

A horseshoe with a U-shaped channel on its under side, and so arranged by a fastening device to have placed a spike or flat-shaped calkings.

A device for stopping runaway horses, consisting of a strap running down the median line of the face, and dividing in two branches just above the nostrils, with a piece of elastic material and a pad to each branch, and a special rein attached to each strap by which pressure on the nostrils and their openings may be made when desired.

An animal-poke, consisting of a yoke and spiral wire extensions at the top and sides.

A nailless horseshoe, consisting of a band raised above the shoe and destined to grasp the hoof and be drawn tight by screws through the ends of the rings of the shoe and fastened to the bands.

A horseshoe with a downwardly extended flange, and spikes extending inwardly from flange to secure a pad within said flange.

A cow-stable appliance, consisting of a pan hung from the ceiling and adjusted with ropes and pulleys and secured to the rear of the cow by straps attached to surcingle and made movable as the cows rise or lie, said pan destined to catch the droppings from the animals.

A rein-holder to be fastened to the dashboard, and a latch arrangement to secure the reins.

A nailless horseshoe, consisting of two parts or halves with an upwardly and inwardly extending flange, destined to engage the outer wall of the hoof, and an inwardly and upwardly extending flange to rest against the concave surface of the sole, pivotally joined at the toe, and with a bolt or screw with lug attachments for regulating adjustment at heels.

